# Effects of Stage Lighting on Visual Comfort at Summer Festivals: A Study in Portugal

**DOI:** 10.3390/healthcare12232441

**Published:** 2024-12-04

**Authors:** Ana Paula Oliveira, Gonçalo Ferreira, Clara Martinez-Perez

**Affiliations:** 1 School of Management, Engineering and Aeronautics, Instituto Superior de Educação e Ciências de Lisboa (ISEC Lisboa), Alameda das Linhas de Torres, 179, 1750-142 Lisboa, Portugal; ana.oliveira@iseclisboa.pt (A.P.O.); 20210593@alunos.iseclisboa.pt (G.F.); 2 Centro de Investigação, Desenvolvimento e Inovação em Turismo (CiTUR)–Polo Estoril Avenida Condes de Barcelona, n.° 808, 2769-510 Estoril, Portugal

**Keywords:** visual comfort, stage lighting, eye health, summer festivals, vision, LED lighting, glare and eye strain

## Abstract

**Background**: The primary objective of this study was to assess the visual comfort and health impacts of stage lighting on attendees at summer festivals. Specifically, the study aimed to evaluate the effects of different types of lighting, including natural, artificial, and stage lighting, on symptoms such as glare, eye strain, tearing, and temporary vision loss. **Methods**: A survey was conducted among attendees of various summer festivals in Portugal. Participants were asked about their perceptions of lighting conditions and the related visual symptoms they experienced. The survey addressed sensitivity to different types of lighting, the impact of smoke on eye discomfort, and potential strategies for improving visual comfort. Data analysis was performed using IBM SPSS^®^ v.27 to explore trends and correlations. **Results**: The findings indicated that cooler stage lighting was associated with a higher incidence of glare, with male participants reporting greater discomfort than females. However, there were no significant differences between gender, age, or refractive status when examining the effects of smoke on symptoms like dry eyes and tearing. Notably, participants aged 19–25 experienced more frequent tearing under stage lighting. Key recommendations included the use of high-quality LED lighting, supported by 44.81% of respondents, and the provision of low-light areas for visual rest, which 37.66% of participants deemed essential. Additional suggestions included minimizing intermittent lights and increasing the awareness of vision protection to improve visual comfort. **Conclusions**: This study highlights the importance of optimizing stage lighting to enhance visual comfort at summer festivals. Festival organizers are encouraged to implement high-quality LED lighting and directional lighting technologies, as well as to create low-light zones for visual rest. Reducing the use of flashing or intermittent lights and providing eye protection information to attendees are also crucial steps to improve the overall visual experience and safeguard eye health at large-scale events.

## 1. Introduction

Cultural events and festivals have been integral to societies since early civilizations, with their origins tracing back to primitive tribes celebrating social and religious events through games and festivities. A key example is the ancient Olympic Games in 776 BC, which combined sports, theater, and music [1]. Festivals not only entertain but also drive social, cultural, and economic transformation. They boost tourism, enhance a destination’s image, create community pride, and generate jobs, stimulating economic growth by extending the tourist season, promoting cultural diversity, and preserving traditions and transmitting them across generations [2,3,4,5]. Their economic impact is critical for sustainable regional development, supporting local businesses and attracting both local and international visitors [4,6]. Urban and rural areas carefully transformed into vibrant cultural hubs at summer festivals are particularly influential in promoting tourism through diverse artistic programs in unique settings [5,6,7,8,9,10,11,12]. These festivals are held worldwide [13,14], and showcase music, dance, theater, and more. The choice of outdoor venues, such as beaches and historic cities, creates a unique synergy between performances, the environment, and the local culture, making the experience immersive and memorable [5,6,11,15,16]. As these festivals grow in popularity, the use of stage artificial lighting plays a crucial role in amplifying the atmosphere. Bright lights, strobe effects, and laser beams are strategically employed to captivate audiences, further elevating the immersive experience by adding excitement and intensity to performances.

However, while lighting enhances the festival environment, it also brings concerns regarding the potential impact on festivalgoers’ vision. The prolonged exposure to high-intensity lights can lead to temporary visual discomfort, including eye fatigue, headaches, and mild dizziness [17,18]. This is due to the human eye’s struggle to adapt to bright, flashing lights for extended periods. Moreover, the contrast between the intense stage lighting and the surrounding darkness often causes rapid changes in light exposure, resulting in symptoms like temporary blurriness or afterimages when viewers shift their gaze from the stage to the darker areas around them [19,20].

Of particular concern is the widespread use of blue light (wavelengths between 400 and 495 nm) emitted by LEDs, which has been linked to various eye health issues [21,22]. Recent studies suggest that prolonged exposure to blue light can damage retinal cells, increasing the risk of age-related macular degeneration [23]. Additionally, this exposure can lead to eye strain and visual discomfort, with symptoms such as dry eyes, headaches, and blurred vision [24]. Another critical impact of intense festival lighting is the disruption of circadian rhythms. Artificial lighting at night interferes with melatonin production, a hormone essential for sleep regulation, raising the risk of sleep disorders and other health issues [22,25,26,27]. Research also suggests that prolonged exposure to artificial light may be linked to an increased risk of chronic diseases, including obesity, diabetes, and certain cancers, such as breast and prostate cancer [28,29,30,31]. Strobe lights, in particular, are known to trigger disorientation or discomfort due to their rapid pulsating nature, which forces the eyes to repeatedly adjust to fluctuating light levels. For individuals predisposed to photosensitive epilepsy, strobe lights can trigger seizures [17,32,33].

Furthermore, the artificial lighting used during festivals can have profound environmental consequences. Bright, intense light disrupts the natural behaviors of wildlife, interfering with migration, reproduction, and feeding patterns across various species. Research has shown that light pollution can disorient migratory birds, draw insects into dangerous environments, and negatively impact both aquatic and terrestrial ecosystems [34,35,36,37].

These potential health and environmental risks underscore the need for effective strategies to mitigate the negative impacts of artificial lighting at festivals. Given the cultural and economic significance of summer festivals, it is crucial for organizers to adopt strategies that safeguard attendees’ well-being while minimizing environmental harm.

**Figure 1:**
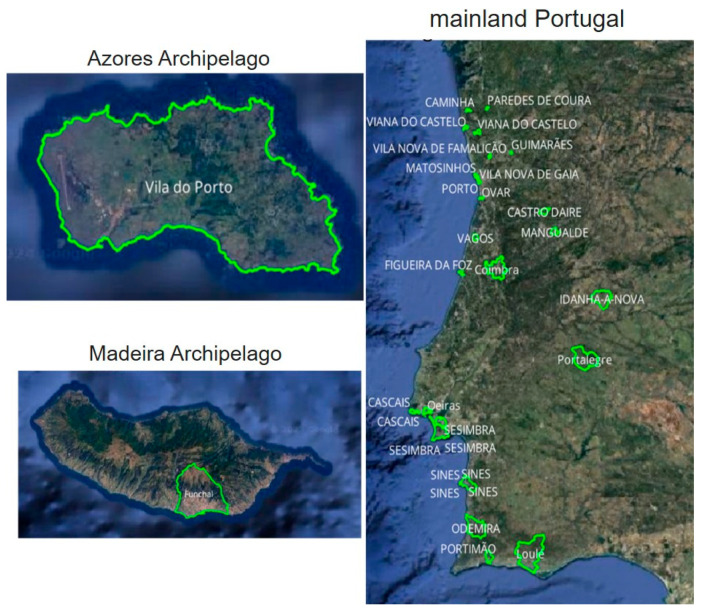
Map of Portugal—mainland Portugal, Azores Archipelago, and Madeira Archipelago—highlighting in green the municipalities that hosted summer festivals in 2023. Source: adapted from Ferreira [40].

Since the late 1960s, summer festivals have been a cultural cornerstone in Portugal, with the pioneering Vilar de Mouros festival being the country’s first and leading major music event [38,39]. This iconic festival laid the foundation for the growth of similar events, establishing a tradition that flourished by the 1990s and early 2000s. During this period, Portugal emerged as a premier destination for modern music festivals, hosting internationally renowned artists and attracting global attention [39]. Today, events like NOS Alive, Super Bock Super Rock, MEO Sudoeste, MEO Marés Vivas, and Boom Festival draw tens of thousands of attendees annually, offering diverse and unforgettable experiences across the country [40]. Notably, approximately 70% of these festivals take place between 15 June and 15 September [39], with Portugal now hosting 33 summer festivals each year (Figure 1).

These festivals play a pivotal role in shaping Portugal’s cultural and musical identity [39,41]. Beyond showcasing world-class performers, they preserve cultural traditions and significantly drive tourism. In 2023, Portuguese festivals drew an impressive 2.1 million attendees, accounting for 7.9% of the country’s summer tourism [42]. Major events like NOS Alive (165,000 attendees), Boom Festival (39,000 participants), and MEO Sudoeste (over 249,000 festivalgoers) exemplify their popularity [40]. Municipalities hosting these festivals have experienced remarkable tourism growth: Caminha, home to Vilar de Mouros, recorded a 200% increase in visitors from 2015 to 2023, while Figueira da Foz, host of RFM SOMNII, saw a 150% rise, and Idanha-a-Nova, the site of Boom Festival, reported 130% growth [40].

Economically, these festivals are vital, stimulating local industries such as hospitality, dining, and retail, while elevating Portugal’s status as a top international tourist destination. Despite their success, challenges remain. Sustainability, safety, and innovation are critical for maintaining momentum. To stay competitive, festivals must embrace new technologies, diversify lineups, and craft unique experiences that distinguish them in a crowded market. Additionally, prioritizing attendees’ well-being will be essential to ensuring that these events remain safe, relevant, and enjoyable for years to come.

This study focuses on the well-being of festivalgoers, specifically addressing the quality of their vision. Its primary objective is to examine the harmful effects of intense lighting on the eye health of attendees at Portuguese festivals. Additionally, the study aims to propose practical solutions to mitigate these risks, ultimately enhancing the safety and overall well-being of festival participants.

## 2. Materials and Methods

### 2.1. Survey

A survey was created using Google Forms^®^, addressed to the Portuguese population. It was distributed through various channels, including social networks such as Facebook, Instagram, and Twitter. To enhance its reach, the survey was shared in festival-related groups and pages and promoted via targeted paid advertisements. Additionally, it was disseminated through the authors’ personal networks and the databases of the Higher Institute of Education and Sciences of Lisbon (ISEC Lisboa). Collaborations with festival organizers further supported its distribution by sharing the survey link at summer 2024 events in Portugal, utilizing direct interactions with attendees and email newsletters. The survey remained accessible from June to September 2024. Participants were not compensated for their responses, and there were no inclusion or exclusion criteria.

The survey comprised 18 questions, with 1 being a closed-ended type, and it was divided into five distinct sections (Supplementary Materials):

(1) General information;
(2) Participation in a summer festival;
(3) Visual Conditions;
(4) Perception about the lights of the festival;
(5) Proposals for improvements.

**Figure 2:**
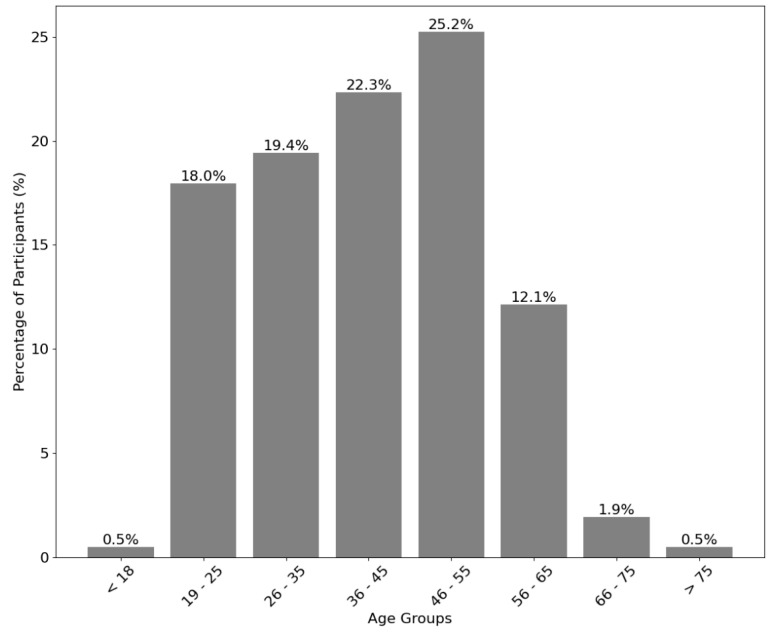
Age distribution of the study participants.

A total of 206 individuals participated in this study by completing the survey. Of these, 154 participants (74.8%) reported having attended a summer festival in Portugal, while 52 participants (25.2%) had not. The gender breakdown revealed that 113 participants (54.9%) identified as female, 92 participants (44.7%) identified as male, and 1 participant (0.5%) preferred not to disclose their gender. Figure 2 illustrates the age group distribution of the participants, with the majority (47.5%) falling within the 36 to 55 age range. In contrast, the <18 and >75 age groups had the lowest representation, each accounting for just 0.5% of respondents.

### 2.2. Statistical Analysis

The survey results were analyzed using IBM SPSS^®^ Statistics v.27. To assess the normality of the data, the Shapiro–Wilk test was applied. Given that most of the data did not follow a normal distribution, non-parametric tests were used for the analysis. Specifically, the Kruskal–Wallis test and Mann–Whitney U test were employed to compare quantitative variables, such as age groups and lighting perception. The Chi-square test was used to analyze differences between categorical variables, such as gender and refractive status in relation to sensitivity to light and visual symptoms. All tests were conducted at a significance level of 0.05. A three-way analysis of variance (ANOVA) was conducted to explore the interaction between gender, age, and visual condition on sensitivity to different types of light (natural, artificial, and stage lighting).

Quantitative data were summarized using the mean and standard deviation for normally distributed data, and the median and interquartile range (IQR) for non-normally distributed data.

All ethical considerations pertinent to the study were strictly followed. Furthermore, the survey was conducted anonymously, adhering fully to the Privacy and Personal Data Protection Policy, as stipulated by Regulation (EU) No. 2016/679 of the European Parliament and the Council of 27 April [43].

## 3. Results and Discussion

### 3.1. Visual Conditions

Among the festival attendees, 51 participants (33.1%) identified as emmetropic, 35 (22.7%) reported having astigmatism, 35 (22.7%) reported myopia, 13 (8.4%) reported hyperopia, and 5 participants (3.2%) reported presbyopia.

**Figure 3:**
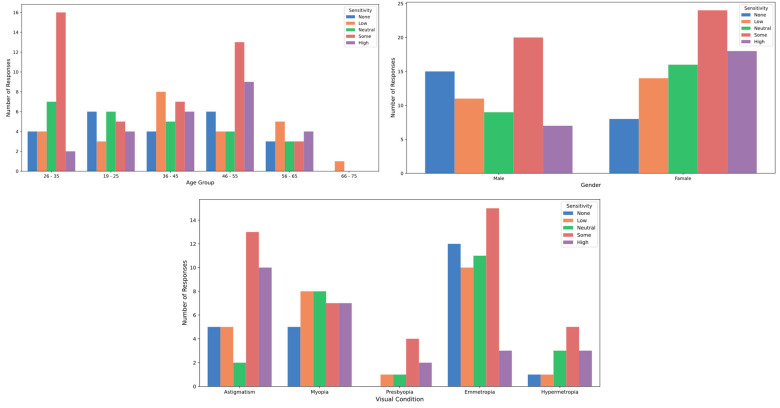
Sensitivity to natural light by age, gender and visual condition.

Table 1 details participants’ sensitivity to various types of light. Regarding natural light, 17.5% of participants reported high sensitivity, while 28.6% exhibited moderate sensitivity. Differences in sensitivity to natural light were analyzed across gender and visual conditions. Women showed significantly higher sensitivity (3.38 ± 1.29, median = 4.0, IQR = [2.0]) compared to men (2.89 ± 1.38, 3.0 [2.0]; *p* = 0.0379; Cohen’s D = 0.36). Sensitivity also varied significantly by visual condition (*p* = 0.0375; Eta Square = 0.083). Participants with astigmatism (3.51 ± 1.42; 4.0 [3.0]), hyperopia (3.62 ± 1.19, 4.0 [1.0]), and presbyopia (3.88 ± 0.99, 4.0 [0.5]) reported higher sensitivity compared to emmetropes (2.75 ± 1.28, 3.0 [2.0]) and myopes (3.08 ± 1.36, 3.0 [2.0]). No significant differences were found with respect to age (*p* > 0.05; Eta Square = 0.031) (Figure 3). Although natural light exposure is typically less harmful than artificial light, it can still impact vision, particularly due to blue light. Blue light, which plays a key role in regulating circadian rhythms, can also cause prolonged visual discomfort. Research indicates that even blue light in natural settings can contribute to visual fatigue and disrupt circadian functions, negatively affecting sleep and overall well-being [15,44].

**Figure 4:**
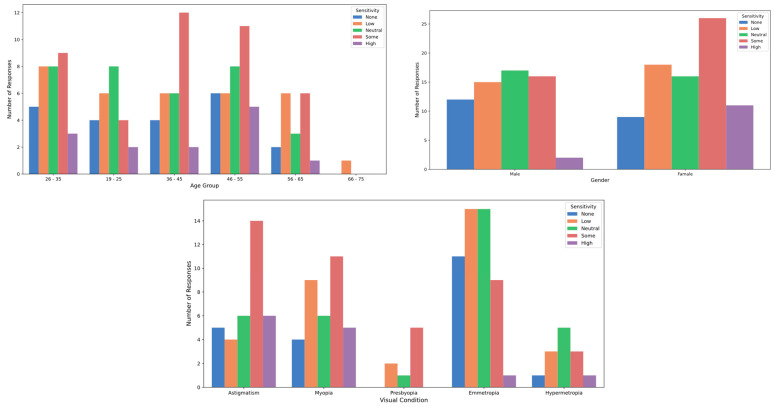
Sensitivity to artificial light by age, gender and visual condition.

On the other hand, 9.1% of respondents reported high sensitivity to artificial light, while 27.3% indicated moderate sensitivity. Differences in sensitivity to artificial light were analyzed across gender and visual conditions. Women showed significantly higher sensitivity (3.15 ± 1.24, 3.0 [2.0]) compared to men (2.70 ± 1.14, 3.0 [2.0]; *p* = 0.0295; Cohen’s D = 0.38). Sensitivity also varied significantly by visual condition (*p* = 0.0227; Eta Square = 0.094). Participants with astigmatism (3.34 ± 1.30, 4.0 [1.50]), presbyopia (3.38 ± 0.92, 4.0 [1.25]), and myopia (3.11 ± 1.28, 3.0 [2.0]) reported higher sensitivity compared to emmetropes (2.49 ± 1.08, 2.0 [1.0]) and hyperopes (3.00 ± 1.08, 3.0 [2.0]). No significant differences were found with respect to age (*p* > 0.05; Eta Square = 0.025) (Figure 4). This aligns with studies showing that prolonged exposure to artificial light sources, especially LEDs and those emitting blue light, can negatively impact ocular health over time [45,46]. Blue light has been linked to reduced contrast sensitivity, increased light scattering, and the appearance of halos and glare, all of which significantly impair night vision and contribute to visual discomfort [15]. Additionally, extended exposure to artificial light has been associated with a higher risk of age-related macular degeneration and other eye conditions, such as computer vision syndrome [44].

**Figure 5:**
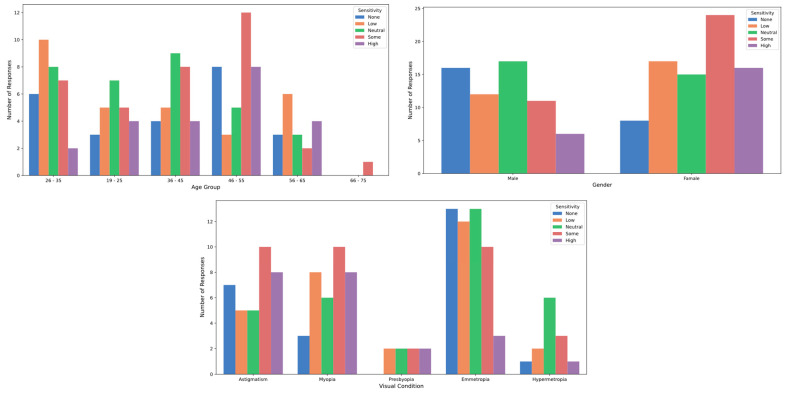
Sensitivity to stage light by age, gender, and visual condition.

Regarding stage lighting, 14.3% of participants reported high sensitivity, while 23.4% experienced moderate sensitivity. Differences in sensitivity to stage lighting were analyzed across gender and age, with significant differences found in both categories, but not by visual condition (*p* > 0.05; Eta Square = 0.073). Women showed significantly higher sensitivity (3.29 ± 1.28, 3.5 [2.0]) compared to men (2.68 ± 1.31, 3.0 [2.5]; *p* = 0.0070; Cohen’s D = 0.47). Sensitivity also varied significantly by age (*p* = 0.0472; Eta Square = 0.035). Participants aged 66–75 years reported the highest sensitivity (4.00, 4.0 [0.0]), followed by those aged 46–55 years (3.25 ± 1.48, 4.0 [2.0]). Younger groups, such as those aged 19–25 years, showed lower sensitivity (3.08 ± 1.28, 3.0 [2.0]) (Figure 5). Stage lights, designed to produce dramatic visual effects, are known to cause glare, eye strain, and temporary visual disruption. Studies have documented that strobe lights and lasers used in performances can cause significant visual discomfort, including blurred vision, flashes, and difficulty adapting when viewers shift from brightly lit areas to darker spaces [15,44]. Moreover, blue light in these setups has been linked to long-term retinal risks, including increased oxidative stress, which can lead to ocular cell damage [47].

When performing a three-way ANOVA, no statistically significant differences were found in sensitivity to natural, artificial, or stage lighting based on gender, age, or visual condition, nor in the interactions between these factors (*p* > 0.05).

### 3.2. Perception About the Festival Lights

**Figure 6:**
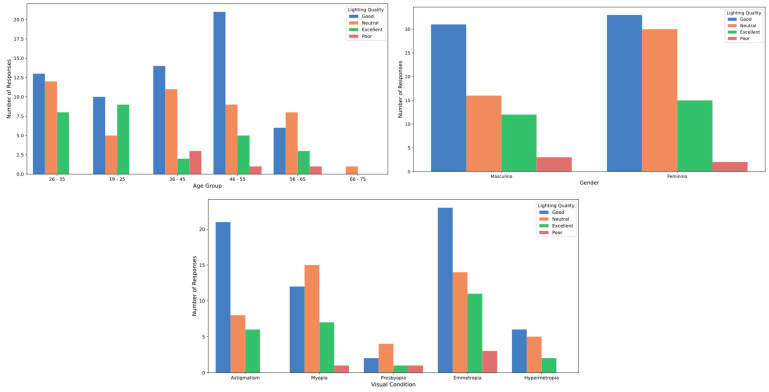
Lighting quality perception by age, gender and refractive status.

In examining the variable related to the quality of stage lighting at festivals, the results show that the mean and standard deviation of participants’ overall perception is 3.81 ± 0.81, on a scale from 1 to 5. The median score was 4.0, with an interquartile range (IQR) of 1.0. No significant differences were observed in the perception of lighting quality between male and female participants, nor were there differences based on refractive status (*p* > 0.05; Cohen’s D = 0.49) (Figure 6). However, a significant difference was noted in lighting quality perception across age groups (*p* = 0.022) (Figure 6), indicating that evaluations of stage lighting vary by age. Participants aged 19 to 25 had a mean score of 4.19 (SD = 0.75), a median of 4.0, and an IQR of 1.0. In contrast, the 36 to 45 age group reported a mean score of 3.5 (SD = 0.78), a median of 4.0, and an IQR of 1.0 (*p* = 0.02597; Cohen’s D = 0.90). Studies on light perception, particularly regarding blue light, have shown that age significantly affects visual sensitivity. As people grow older, the crystalline lens of the eye becomes less transparent and develops a yellowish tint, reducing its effectiveness in filtering blue light. This diminished filtering ability increases the risk of glare and visual sensitivity in older adults, often leading to substantial visual discomfort [22,23]. Additionally, photosensitive retinal ganglion cells, which contain melanopsin and regulate circadian rhythms as well as non-visual responses to light, are especially sensitive to blue light wavelengths around 480 nm. As these cells’ responsiveness declines with age, individuals experience heightened glare sensitivity and a reduced capacity to adapt to changes in lighting conditions [22]. This decline is also linked to sleep disturbances in older adults, as their circadian rhythms are increasingly disrupted by exposure to artificial blue light [23].

When assessing the aesthetic perception of the lights used on festival stages, 43.51% of participants (n = 67) found the lights to be aesthetically pleasing, while 36.36% (n = 56) expressed an indifferent attitude toward them. Conversely, 20.13% of respondents (n = 31) reported that the lights were uncomfortable.

**Figure 7:**
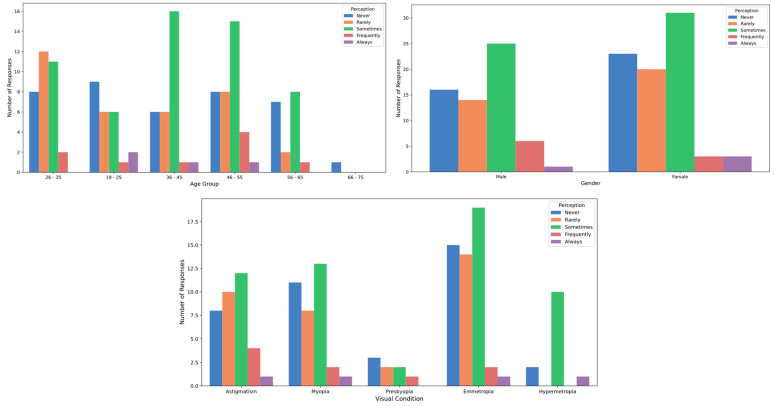
Glare perception by age, gender and refractive status.

**Figure 8:**
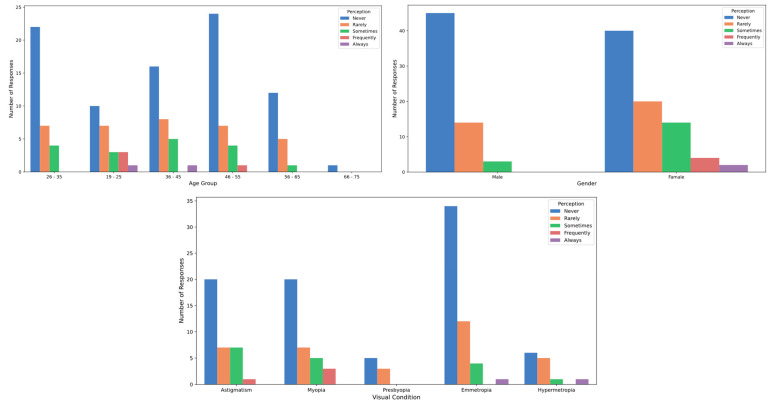
Tearing perception by age, gender and refractive status.

**Figure 9:**
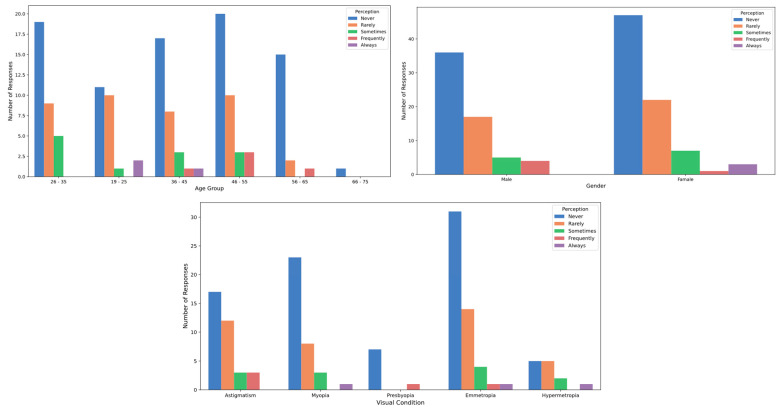
Vision loss perception by age, gender and refractive status.

Table 2 presents the participants’ perceptions of the effects of stage lighting. Regarding the sensation of glare, slight differences were observed between genders: 41.18% of men reported experiencing glare “sometimes,” compared to 38.82% of women. Additionally, 8.82% of men reported feeling glare “frequently”, while only 3.53% of women reported the same frequency. However, these differences were not statistically significant (*p* > 0.05; Cohen’s D = 0.11). In terms of age, individuals in the 36 to 45 age group reported a higher incidence of experiencing glare “sometimes” (53.33%) compared to other age groups, although this difference was not statistically significant (*p* > 0.05; Cohen’s D = 0.21) (Figure 7). When it came to tearing, a higher proportion of participants aged 19 to 25 reported experiencing this symptom “frequently” (11.54%) compared to other age groups, yet, again, no significant differences by age were found (*p* > 0.05; Cohen’s D = 0.32) (Figure 8). Concerning temporary vision loss, the 56 to 65 age group had the highest percentage of individuals who reported never experiencing this symptom (80.00%), but the differences across age groups were not statistically significant (*p* > 0.05; Cohen’s D = 0.44). Finally, individuals with hyperopia reported a greater incidence of “rarely” experiencing temporary vision loss (38.46%) compared to those with myopia (22.86%), but this difference was also not statistically significant (*p* > 0.05) (Figure 9).

With regards to the perception of stage lighting quality between male and female participants, Shaqiri et al. [48] identified sex-related differences in visual perception, with men generally outperforming women in tasks such as motion detection, while women showed greater sensitivity to color and certain visual stimuli. Comparing our findings with those of Shaqiri et al., we observed that the differences in sensitivity to natural, artificial, and stage lighting are consistent with the previously reported gender variations. Specifically, women showed higher sensitivity to natural and artificial lighting, which may be influenced by hormonal factors affecting retinal sensitivity, aligning with Shaqiri et al.’s suggestion of sex-based physiological differences. On the other hand, men reported more discomfort with stage lighting, possibly due to differences in visual adaptation to intense, rapidly changing light, as highlighted by Shaqiri et al. [48].

In terms of similarities, both our study and that of Shaqiri et al. [48] found no significant gender differences in glare sensitivity, tearing, and vision loss, suggesting that these aspects of visual discomfort are comparable between males and females. These findings indicate that while certain aspects of visual perception, such as sensitivity to specific light types, exhibit gender-based differences, other elements like glare and tearing do not significantly differ. These variations and similarities highlight the complex nature of sex-related differences in visual perception, which could be further investigated under controlled lighting conditions to better understand the underlying mechanisms.

Prolonged exposure to intense light sources, particularly blue-rich lighting during events such as festivals, has significant side effects on visual health, many of which are associated with the increasing prevalence of digital eye strain and glare. Various studies have highlighted that blue light with short wavelengths (400–500 nm) induces photochemical damage to photoreceptors and the retinal pigment epithelium due to increased oxidative stress. This mechanism, well-documented in in vitro studies, is associated with degenerative changes in the retina and a higher prevalence of related eye diseases, such as age-related macular degeneration (AMD) [49,50].

Moreover, prolonged exposure to this type of lighting affects the ocular surface, contributing to dry eye symptoms. This effect is partly due to a reduced blinking rate in environments with high light intensity and stroboscopic effects, leading to symptoms such as burning, blurred vision, and a sensation of visual fatigue) [50]. In large-scale events, where the use of intermittent light effects is common, more immediate impacts are also observed, such as glare and difficulty adapting to darkness, which may compromise the safety and visual experience of attendees.

On the other hand, recent studies have questioned the effectiveness of strategies such as blue light filters in reducing these effects. While these tools have been proposed to mitigate glare and improve visual comfort, systematic reviews suggest their impact on reducing visual fatigue and improving sleep quality is limited and, in many cases, not clinically significant [51,52,53,54,55]. However, mild side effects, such as headaches or discomfort when using glasses with filters, underscore the need for the careful design of these interventions to ensure their acceptance and effectiveness.

Given the complexity of the problem, it is essential to implement practical solutions to mitigate these side effects. Suggested strategies include using adjustable lighting technologies, strategically distributing visual rest areas, and disseminating information on eye protection, such as wearing sunglasses or specific filters. These measures would not only benefit festival attendees but also address broader issues related to chronic exposure to artificial light sources, which are increasingly present in our daily lives.

**Figure 10:**
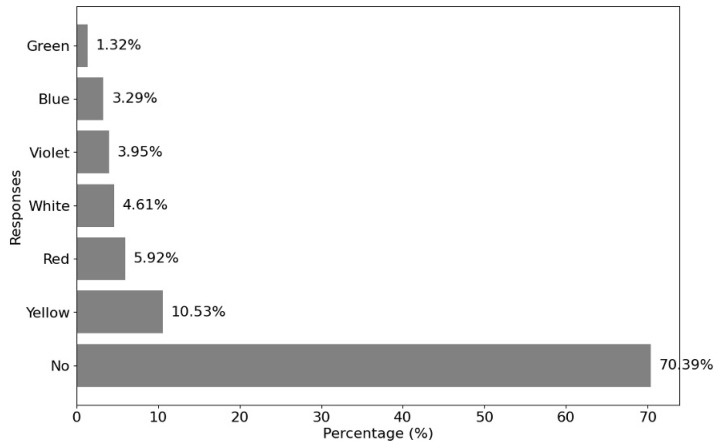
Impact of light colors on symptoms.

Figure 10 illustrates the impact of different colors on visual symptoms. When comparing these results with previous studies, several similarities are observed regarding how both color and temperature of lighting influence visual discomfort. Some studies have indicated that higher color temperatures, such as 5700 K, create a brighter environment but do not necessarily enhance visual comfort [56,57]. In fact, lower temperatures, like 4000 K, are often more effective at reducing glare and improving visual comfort in LED-lit settings [56,57].

Our results support this observation, revealing that participants exposed to cooler lighting reported a greater incidence of glare, with men experiencing discomfort more frequently than women. This finding aligns with existing research suggesting that men may be more sensitive to intense lighting conditions [23,45]. Additionally, the relationship between age and glare sensitivity has been well documented [58,59]. As individuals age, the lens of the eye becomes less transparent and more opaque, affecting its ability to filter high-energy blue and violet light. This leads to a higher propensity for glare in older adults under intense lighting conditions [58,59]. In this study, the 36 to 45 age group exhibited a higher incidence of glare, reflecting this trend, although the differences were not statistically significant.

Exploring the effects of color on visual symptoms, numerous studies have highlighted how different wavelengths, such as blue, red, and violet, can intensify symptoms like glare, tearing, and temporary vision loss [15,23,45]. Blue light, in particular, is linked to increased light scattering, which can create halos and diminish visual quality. The use of intense violet and blue lights often leads to eye fatigue due to their high energy levels, a phenomenon commonly observed in stage lighting environments where these colors are frequently utilized [15,23,45]. Violet light, being the most susceptible to scattering, generates more glare and can significantly reduce contrast sensitivity [15]. Conversely, while red light is less phototoxic, it can still cause visual discomfort in high-contrast settings, especially during transitions from bright light to darkness. Research indicates that red light can slow visual adaptation, which may account for the blurred vision reported by participants in our study [15,23]. In contrast, colors such as green and yellow tend to cause fewer issues regarding glare and visual fatigue, as they are perceived with less intensity compared to blue and violet light [15].

Table 3 presents the results of participants’ perceptions of the effects of smoke on their eyes. No significant differences were found based on gender, age, or refractive status concerning symptoms of dry eyes (Cohen’s D = 0.51), tearing (Cohen’s D = 0.38), and a gritty sensation (Cohen’s D = 0.44) (all *p* > 0.05). This finding aligns with existing research indicating that exposure to smoke, whether from domestic sources, wildfires, or tobacco, affects all demographic groups uniformly [60,61,62]. Previous studies have demonstrated that wildfire smoke contains fine particulate matter (PM2.5) and volatile organic compounds (VOCs), which can lead to eye irritation, dryness, and tearing, regardless of age or gender [60,61]. Indoor exposure to tobacco smoke and smoke from biomass fuels, such as wood or charcoal, has also been associated with widespread eye irritation across diverse populations. For instance, a study conducted in Guatemala found that over 60% of women exposed to smoke from wood-burning stoves reported persistent eye irritation [60]. Similarly, studies on vehicle traffic pollution and exposure to wildfire smoke have identified that the impact on ocular health, such as the development of conjunctivitis or dry eye syndrome, does not vary by individual factors such as gender or refractive status, affecting all exposed groups equally [61,62].

### 3.3. Improvement Proposals

Table 4 indicates that 37.66% of respondents considered the establishment of areas with reduced lighting for visual rest to be important, with an additional 14.29% deeming it very important. This perspective aligns with best practices in lighting design, as research suggests that balancing brightly lit areas with shaded or dimly lit spaces can effectively reduce eye strain, allowing attendees to recuperate from the effects of intense stage lighting [63].

Additionally, 44.81% of respondents rated the use of high-quality LED lighting as important, while 11.69% regarded it as very important. The preference for high-quality LED lighting in event production is well justified, as these systems offer significant advantages, including energy efficiency, longevity, and lower heat emission—all factors that enhance visual comfort and minimize glare. Festivals are increasingly adopting LED downlights, which are favored for their directional lighting capabilities and dimmability, allowing precise adjustments to meet the unique requirements of each event [56]. Moreover, advanced LED systems provide precise control over light intensity and color, enabling the creation of visually engaging environments while minimizing harsh brightness and reducing eye strain.

Further recommendations from the study highlight the importance of implementing lighting control technology, as endorsed by 45.45% of respondents, and employing directional, structured lighting, which is considered important by 46.75%. These practices align with industry standards aimed at enhancing visual performance and comfort. Directional lighting is especially vital for minimizing unnecessary light spill and concentrating illumination where it is most needed, thereby improving overall visual comfort [63].

Additional suggestions include minimizing the use of intermittent lighting, deemed important by 31.82% of respondents, and increasing shaded areas, which 37.01% rated as important. These findings reflect broader concerns regarding the potential adverse effects of flickering lights, which can contribute to eye fatigue and discomfort, particularly during extended exposure [63,64].

Lastly, distributing sunglasses—important to 27.27% of respondents—and providing more information on vision protection—supported by 36.36%—are practical measures that align with event safety guidelines. These initiatives are especially crucial in outdoor settings, where minimizing direct exposure to intense light sources is essential for attendees’ comfort [64].

Other recommendations for festival organizers to optimize stage lighting to enhance visual comfort include the use of dynamic dimming systems, which adjust lighting in real time to maintain visual balance as the surrounding environment changes. Strategically positioning light fixtures to minimize direct glare by angling them away from attendees’ line of sight can also help reduce discomfort. Additionally, incorporating diffused lighting and frosted filters can soften the intensity of strobe lights and lasers, providing an engaging visual experience without overwhelming the audience. By combining these strategies with input from lighting designers and festivalgoers, it is possible to create a lighting environment that is both immersive and comfortable. Furthermore, organizers should actively promote eye care among festival attendees. Simple recommendations, such as periodically looking away from intense light sources or briefly closing their eyes during visually demanding performances, can help reduce eye strain. Combining thoughtful lighting design with practical guidance ensures a safer and more enjoyable festival experience for everyone.

This study has certain limitations that should be acknowledged. First, the sample size of 206 participants, while sufficient for exploratory analysis with an estimated margin of error of 6.8%, does not achieve the 5% margin of error typically desired for population-wide estimates. This limitation has been addressed by focusing on identifying trends and relationships rather than drawing generalized conclusions. Second, the study relies on self-reported data, which may introduce biases such as recall bias or social desirability bias, potentially affecting the accuracy of the responses. Third, the cross-sectional design captures data at a single point in time, limiting our ability to infer causal relationships between stage lighting and reported symptoms. Finally, the geographic scope of the study, conducted exclusively in Portugal, may affect the generalizability of findings to other populations or cultural contexts. Future research with larger, more diverse samples and longitudinal designs is recommended to enhance the robustness and applicability of these findings.

Since our study could be applied to comprehend lighting design techniques to improve visual comfort in public events, the clinical value might be very pivotal. Our results may help inform the development and application of customized lighting arrangements to minimize visual discomfort (glare and tearing) experienced by men and women through the detection of target lighting conditions that differentially affect each gender. This is important regarding making the experience more enjoyable for all attendees and helping to keep them out of harm’s way, whilst catering for sensitive populations who may have medical conditions that are triggered by certain types of lighting. Furthermore, knowledge about the dependence of the response on gender could help in creating guidelines to reduce the risk of vision impairment in environments with high-intensity artificial light. 

## 4. Conclusions

The survey results offered valuable insights into how different lighting setups at summer festivals affect attendees’ visual comfort. A significant portion of participants reported discomfort, particularly from blue light sensitivity, glare, eye strain, and temporary vision disturbances.

The most common issues were linked to the intense lighting used during stage performances, which caused moderate-to-high levels of discomfort. Notably, 33.1% of festivalgoers with refractive errors experienced heightened sensitivity to artificial and stage lighting. Although participants across age and gender reported similar symptoms, younger attendees (aged 19–25) experienced higher rates of tearing under stage lights. However, these differences were not statistically significant. Recommendations based on the findings include the strategic use of lighting technologies, such as high-quality LED systems, which offer better control over brightness and reduce the harmful effects of blue light. Additionally, creating designated areas with reduced lighting for visual rest was strongly supported by respondents (37.66% rated this as important), aligning with industry practices for reducing visual strain in high-intensity lighting environments. Another effective measure would be limiting the use of strobe and flickering lights, which can exacerbate discomfort and, in some cases, trigger photosensitive reactions in certain individuals.

While the aesthetic appeal of festival lighting was acknowledged, the practical need to prioritize visual health was emphasized. Organizers should consider strategies like promoting awareness, providing sunglasses, and offering shaded rest areas to protect attendees’ vision. These measures, supported by current lighting and health research, can create a safer and more comfortable experience for festivalgoers. By integrating these approaches, festival organizers can address the visual health concerns highlighted in this study, while preserving the vibrant, immersive atmosphere that defines large-scale events.

## Figures and Tables

**Table 1:** Sensitivity to different types of light.

	A lot	Some	Neutral	Little	None
Natural Light	27 (17.5)	44 (28.6)	28 (18.2)	30 (19.5)	25 (16.2)
n (%)					
Artificial Light	14 (9.1)	42 (27.3)	39 (25.3)	36 (23.4)	23 (14.9)
n (%)					
Stage Light	22 (14.3)	36 (23.4)	37 (24.0)	33 (21.4)	26 (16.9)
n (%)					

**Table 2:** Perception of stage lighting effects and symptoms.

	Never n (%)	Rarely n (%)	Sometimes n (%)	Frequently n (%)	Always n (%)
Glare	42 (27.27)	38 (24.68)	61 (39.61)	9 (5.84)	4 (2.6)
Tearing	92 (59.74)	39 (25.32)	17 (11.04)	4 (2.6)	2 (1.3)
Temporary vision loss	90 (58.44)	43 (27.92)	13 (8.44)	5 (3.25)	3 (1.95)

**Table 3:** Perception of the effects of smoke on the eyes.

	Never n (%)	Rarely n (%)	Sometimes n (%)	Frequently n (%)	Always n (%)
Dry eye	80 (51.95)	27 (24.03)	26 (16.88)	7 (4.55)	4 (2.60)
Tearing	84 (54.55)	39 (25.32)	22 (14.29)	6 (3.90)	3 (1.95)
Gritty sensation	99 (64.29)	30 (19.48)	19 (12.34)	4 82.60)	2 (1.30)

**Table 4:** Recommended measures for festival organizers to enhance visual comfort.

	Not Important n (%)	Slightly Important n (%)	Indifferent n (%)	Important n (%)	Very Important n (%)
Create areas with reduced lighting for visual rest	15 (9.74%)	20 (12.99%)	39 (25.32%)	58 (37.66%)	22 (14.29%)
Use high-quality LED lighting	7 (4.55%)	8 (5.19%)	52 (33.77%)	69 (44.81%)	18 (11.69%)
Use lighting control technology	6 (3.90%)	8 (5.19%)	50 (32.47%)	70 (45.45%)	20 (12.99%)
Use directional and structured lighting	5 (3.25%)	9 (5.84%)	54 (35.06%)	72 (46.75%)	14 (9.09%)
Reduce the use of intermittent lights	5 (3.25%)	12 (7.79%)	45 (29.22%)	49 (31.82%)	43 (27.92%)
Create more shaded areas	8 (5.19%)	18 (11.69%)	47 (30.52%)	57 (37.01%)	24 (15.58%)
Strategically place lights and diffusers to prevent glare	7 (4.55%)	10 (6.49%)	47 (30.52%)	68 (44.16%)	22 (14.29%)
Distribute sunglasses and eye protection	21 (13.64%)	26 (16.88%)	38 (24.68%)	42 (27.27%)	27 (17.53%)
More information and guidance on how to protect vision	10 (6.49%)	15 (9.74%)	38 (24.68%)	56 (36.36%)	35 (22.73%)

## Data Availability

Data are contained within the article and Supplementary Materials.

## Supplementary Materials

The following supporting information can be downloaded at: https://www.mdpi.com/article/10.3390/healthcare12232441/s1, Survey.
