# Supplementary material for: Effects of Stage Lighting on Visual Comfort at Summer Festivals: A Study in Portugal
Source: Healthcare (Basel). 2024 Dec 4;12(23):2441. doi: 10.3390/healthcare12232441 (PMC11641230; doi:10.3390/healthcare12232441)
Supplement: Supplementary file 1 [file healthcare-12-02441-s001.zip › healthcare-3318064-supplementary.pdf]

Supplementary Materials - SURVEY

INFORMED CONSENT

I consent to the storage and use of the data I provide, exclusively for research purposes related to this topic, with full assurance that my anonymity will be maintained at all times.

Yes

No

GENERAL INFORMATION

1. Gender

Female

Male

I prefer not to say

Other

2. Age

< 18 years old

19-25 years old

26-35 years old

36-45 years old

46-55 years old

56-65 years old

66-75 years old

> 76 years old

3. Birthplace (insert the municipality where you were born)

PARTICIPATION IN A SUMMER FESTIVAL

4. Have you ever participated in a Summer Festival in Portugal?

Yes

No

5. How did you participate in the festival?

Festivalgoer

Organizer

Artist

6. Which festival(s) have you attended in the last 5 years? (indicate the name(s) of the festival(s))

____

7. What time of day did you participate in the festival(s)?

During the day, with natural light (sun)

At night, with artificial lights

In both periods (day and night)

VISUAL CONDITIONS

8. What is your visual condition?

Myopia

Hyperopia

Astigmatism

I don't know

I have no conditions

9. Do you use any visual correction methods?

Glasses

Contact lenses

Both

I don't need to use any correction method

10. During the festival, what method is used for visual correction?

Glasses

Contact lenses

Both

None

I don't need to use any correction method

11. In general, how sensitive are you to?

	None	Little	Neutral	Some	Many
natural light?	◯	◯	◯	◯	◯
artificial light?	◯	◯	◯	◯	◯
stage light?	◯	◯	◯	◯	◯

PERCEPTION ABOUT THE LIGHTS OF THE FESTIVAL

12. In general, how would you describe the quality of the stage lighting at the festivals you've attended? (select a number between 1 to 5)

Very Good 1 2 3 4 5 Very Good

13. Are the lights used on festival stages, in your opinion, aesthetically pleasing or annoying?

They are pleasant

Bother

Indifferent

14. Regarding stage lights, have you ever felt

	Never	Rarely	Sometimes	Often	Always
confused?	◯	◯	◯	◯	◯
tearing up?	◯	◯	◯	◯	◯
no momentary vision?	◯	◯	◯	◯	◯

15. Do any of the above symptoms worsen with a certain color of light?

No

Yes, with red light

Yes, with yellow light

Yes, with blue light

Yes, with green light

Yes, with violet light

16. How does the intensity of stage lighting impact your festival experience?

Increases energy and emotion

Highlights the performances

Increases concern for the well-being of local fauna

Intensifies involvement with the show

Disorientation, making it difficult to move around the festival

Allows you to capture quality photos and videos

Uncomfortable for the eyes and visual fatigue

Highlights the artists and their movements on stage

Need to move away from the stage due to the light being very strong

Other

17. Regarding the smoke from the stage, have you ever felt your eyes

Never	Rarely	Sometimes	Often	Always

dry?	○	○	○	○	○
tearing up?	○	○	○	○	○
gritty sensation?	○	○	○	○	○

PROPOSALS FOR IMPROVEMENTS

18. What measures should festival organizers take to improve visual comfort for festivalgoers?

	Not important	Unimportant	Indifferent	Important	Very important
Create areas with reduced lighting for visual rest	○	○	○	○	○
Use high quality LED lighting	○	○	○	○	○
Use lighting control technology	○	○	○	○	○
Use targeted and structured lighting	○	○	○	○	○
Reduce the use of flashing lights	○	○	○	○	○
Create more shaded spaces	○	○	○	○	○
Strategically place lights and diffusers to prevent glare	○	○	○	○	○
Distribute sunglasses and eye protectors	○	○	○	○	○
More information and guidance on how to protect your vision	○	○	○	○	○
Other	○	○	○	○	○
